# The Potential Role of Glucagon-Like Peptide-1 (GLP-1) Receptor Agonists as a Type of Conservative Treatment of Endometrial Cancer in Women of Reproductive Age: A Review of the Literature and a Call for Study

**DOI:** 10.7759/cureus.69678

**Published:** 2024-09-18

**Authors:** Maria Zoi Bourou, Alkis Matsas, Georgios Valsamakis, Nikolaos Vlahos, Theodoros Panoskaltsis

**Affiliations:** 1 Second Department of Obstetrics and Gynecology, National and Kapodistrian University of Athens Medical School, Aretaieio University Hospital, Athens, GRC

**Keywords:** conservative treatment, endometrial cancer, fertility sparing, glp-1 receptor agonists, t2dm

## Abstract

Endometrial cancer (EC) is among the most common gynecological malignancies in developed countries and its occurrence has been increasing dramatically in the past few years. An in-depth knowledge of the causes of endometrial cancer, such as unopposed estrogen, insulin resistance, and chronic inflammation, has resulted in the suggestion of numerous interventions to decrease the occurrence of this cancer. Recent research has established a connection between obesity and type 2 diabetes mellitus (T2DM) with a higher chance of developing endometrial cancer, suggesting that insulin resistance is a key factor in its onset. Moreover, evidence from both epidemiological and clinical studies indicates that metformin, a drug used to treat diabetes, could possibly help in the prevention of specific types of cancer such as endometrial cancer. The aim of this study is to explore the possible impact of glucagon-like peptide-1 (GLP-1) receptor agonists (RAs) in the non-surgical management of endometrial cancer. GLP-1 has various functions and is produced when nutrients are consumed. Besides promoting the release of insulin, GLP-1 also suppresses the secretion of glucagon and reduces appetite. Moreover, the fact that GLP-1 receptors are found in different organs and tissues such as the brain, lung, pancreas, stomach, heart, and endometrium indicates that GLP-1RAs have multiple functions. Prior research has shown that it triggers apoptosis in endometrial cancer cells. Nevertheless, the precise physiological function of GLP-1 receptors in endometrial cancer still needs to be fully understood.

## Introduction and background

Endometrial cancer (EC) is a commonly seen gynecological cancer, mostly impacting women in the perimenopausal and postmenopausal periods, with approximately a quarter of cases occurring in women of reproductive age. Unexpectedly, less than 5% of EC patients are younger than 40, with over 70% of them not having given birth due to delaying pregnancy in Western cultures [1]. EC is divided into two main categories: Type I and Type II. Type II is associated with advanced patient age and a worse outcome. Most Type I EC patients have increased estrogen production in their fat cells. Having no children and being unable to conceive are also significant factors that increase the risk of endometrial cancer. Fertility-preserving therapies are only suitable for Type I endometrial cancer [2]. Numerous conservative options exist for treating EC, with pharmaceutical drugs like oral progestins, levonorgestrel-intrauterine device (LNG-IUD), gonadotropin-releasing hormone agonist (GnRHa), selective estrogen receptor modulators (SERMs), selective estrogen receptor degraders (SERDs), aromatase inhibitors (AIs), and metformin being the most frequently used. According to the most recent European Society of Gynaecological Oncology (ESGO) recommendations, the best treatment option is a combination of LNG-IUD and oral progestin therapy, which provides a low recurrence rate and a satisfactory pregnancy rate [3]. Anti-estrogen drugs can serve as the main therapy for overweight EC patients or as a backup choice if progestin treatment is ineffective. Metformin's effectiveness in treating EC is due to its influence on glucose metabolism and its ability to interact with endometrial cancer cells, potentially hindering the growth and advancement of cancer [1].

## Review

Materials and methods

A review of literature was conducted to examine the impact of glucagon-like peptide-1 (GLP-1) receptor agonists (RAs) on cancer, with a specific emphasis on their potential adjunctive role in the conservative management of endometrial cancer. The search was carried out on Medline using the keywords glucagon-like-peptide-1 receptor agonists, endometrial cancer, and conservative treatment, in combination with the boolean operators AND or OR. Only articles published in English from 2013 onwards were induced, with the most recent search conducted in April 2024.

Metformin and cancer

Recent studies have found proof that metformin is effective in treating patients with atypical endometrial hyperplasia (AEH) and early endometrial cancer (EEC). Metformin is proven to hinder the proliferation of different types of cancer cells through its impact on glucose metabolism and the phosphatidylinositole-3 kinase-akstrain transforming-mammalian target of rapamycin (PI3K-Akt-mTOR) signaling pathway. Research has shown the importance of AMP-activated protein kinase (AMPK) in controlling intricate molecular processes other than just cellular energy regulation. AMPK is essential in the PI3K-Akt-mTOR pathway, enhancing apoptosis and impacting tumor growth [4,5]. Moreover, metformin has been shown to increase progesterone receptor expression and make resistant cancer cells more sensitive to agents that induce apoptosis. A meta-analysis provided additional evidence for metformin's anti-cancer effects in patients with AEH and EC. Despite some mixed findings, the combination of metformin with progestin has demonstrated promise in reversing AEH and enhancing outcomes for EC patients [6]. Nevertheless, more investigation is required to comprehensively grasp the effects of metformin on endometrial proliferation and its function in treating endometrial hyperplasia [7].

GLP-1RAs

**Figure 1:**
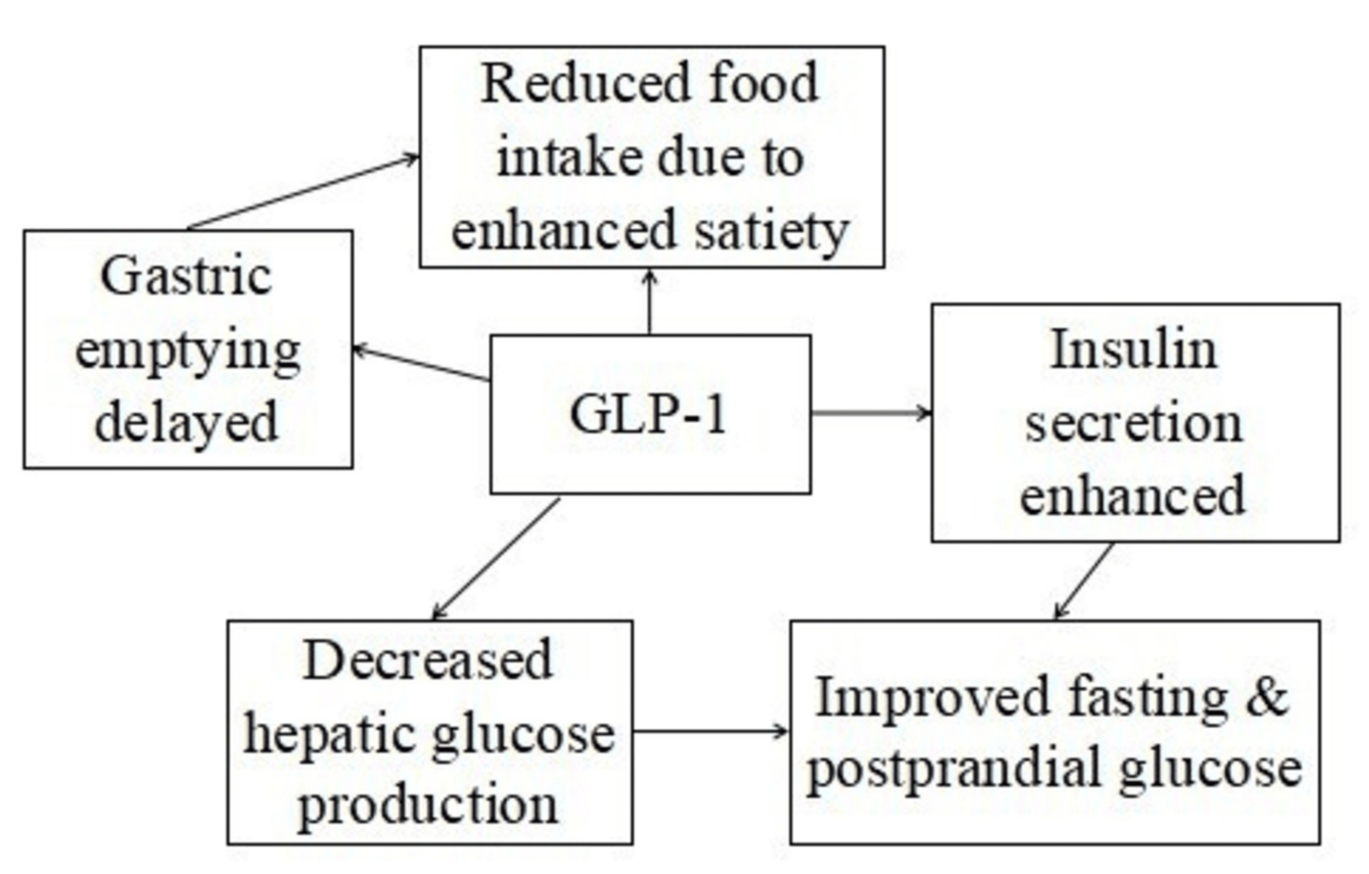
Mechanisms of action of glucagon-like peptide-1 (GLP-1) This figure is an original illustration created by the first author.

GLP-1RAs are injectable drugs that are utilized to decrease glucose levels in adults who have type 2 diabetes mellitus (T2DM). In the United States (US), there are two GLP-1RAs that need to be taken every day (exenatide and liraglutide) and three that need to be taken once a week (albiglutide, dulaglutide, and semaglutide). These medications offer much greater GLP-1 receptor activation than the body's own GLP-1, resulting in better glycemic control and decreased body weight [8]. Stimulating GLP-1 receptors in the pancreas boosts insulin secretion and decreases glucagon secretion, leading to glucose-dependent reactions with a minimal risk of hypoglycemia. Furthermore, stimulation of GLP-1 receptors in both the central nervous system (CNS) and gastrointestinal tract (GIT) leads to a decrease in hunger and a postponement of glucose absorption by slowing down the emptying of the stomach (Figure 1).

GLP-1RAs are suggested as second-line treatment along with other drugs to improve blood sugar management and offset weight increase. Primary care physicians find them to be an important asset in assisting patients with T2DM to reach their treatment goals [8]. Obesity has a strong connection to endometrial cancer, and losing weight is linked to a lower risk because it improves insulin sensitivity and inflammation levels. Yet, it can be difficult to sustain weight loss, potentially resulting in weight fluctuations that increase the risk of endometrial cancer more than consistently being at a higher weight [7].

GLP-1 RAs and polycystic ovary syndrome

Recent literature has explored the relationship between GLP-1RAs and polycystic ovary syndrome (PCOS). PCOS is a prevalent reproductive endocrine disorder that impacts women of childbearing age and is a contributing factor for EC type I [9]. Losing weight is seen as the main approach, and it may enhance PCOS factors. GLP-1RAs have been proven to be successful in treating PCOS either on their own or when used along with other GLP-1RAs. Multiple research studies have pointed out that GLP-1RAs, notably liraglutide, may have an advantage in decreasing body weight and body mass index (BMI) compared to metformin. In contrast, multiple articles have shown that using a combination of GLP-1RA and metformin treatment has produced positive outcomes in improving metabolic and endocrine factors when compared to using just one treatment. GLP-1RAs fall under pregnancy category C and are not advised for use in pregnant women. In clinical trials, the use of GLP-1RAs showed minimal safety concerns, suggesting an acceptable clinical profile. Careful observation and a proper cleansing period are important requirements [10].

T2DM and cancer

Multiple epidemiological studies have shown a clear connection between T2DM and the occurrence of endometrial cancer, hepatobiliary cancer, pancreatic cancer, breast cancer, prostate cancer, and colorectal cancer. The main reason for the higher cancer risk in diabetic individuals is mainly due to elevated levels of insulin in the blood, resulting in a reduction in insulin-like growth factor (IGF) binding proteins. This decrease leads to higher levels of unbound IGF-1 in cells and tissues, which is closely linked to a higher risk of cancer. As a result, individuals diagnosed with T2DM are at a higher risk of developing different types of cancer in comparison to those who do not have the condition. In recent years, researchers have increasingly focused on the potential effects of GLP-1RAs on tumor growth and advancement [11].

GLP-1RAs and endometrial cancer

A recent study conducted by Zhang and colleagues explored the effects of GLP-1RAs on endometrial cancer, using both in vivo and in vitro methods [12]. The research team separated subcutaneous human endometrial cancer cell Ishikawa xenografts in nude mice into control and exenatide-treated groups to investigate if exenatide enhances or hinders the growth of endometrial cancer. Their research uncovered that exenatide has the potential to inhibit the proliferation of endometrial cancer Ishikawa cell xenografts, which is consistent with findings from other recent studies. Furthermore, it was noted that exenatide raised the overall serum GLP-1 concentration, however, there was no significant effect on blood glucose levels, insulin, and IGF-1 levels in the control group. The scientists determined that increased serum GLP-1 levels in the group treated with exenatide resulted in a more potent inhibitory impact on the human endometrial cancer Ishikawa cell xenograft in nude mice. GLP-1R was also found in human endometrial cancer cells, indicating that GLP-1R is highly expressed in both cancerous and non-cancerous endometrial cells. Thus, exenatide may inhibit EC growth through GLP-1R signaling. The research also investigated the IGF-1R/PI3k/Akt/mTOR signaling pathway, which has been studied in many researches and shown to be increased in endometrial cancer. Moreover, in numerous cancer types, mTOR is overexpressed as a result of genetic changes or abnormal activation of PI3K/AKT pathway elements, leading to disruptions in cell proliferation, differentiation, growth, and viability. Zhang and colleagues reported that exenatide inhibits EC cell growth by suppressing phosphorylation, leading to the inhibition of mTOR phosphorylation and promotion of apoptosis. Results from experiments done in a laboratory setting showed that exendin-4 inhibited the growth of EC Ishikawa cells in a manner that depended on the dose and time, leading to apoptosis and increased expression in the Ishikawa cells [12].

Following the results of the prior research, Kanda et al. explored the physiological effects of liraglutide, a GLP-1RA, on Ishikawa endometrial cancer cells. Tissue samples were used by them to examine the pathological functions of GLP-1R in individuals with endometrial cancer. The findings showed that liraglutide, an activator of GLP-1R, increased the levels of GLP-1R in Ishikawa endometrial cancer cells and inhibited the growth of these cells depending on the dosage. They also discovered that liraglutide can trigger the AMPK signaling pathway and promote the buildup of autophagosomes. Additionally, the joint use of liraglutide and 5-aminoimidazole-4-carboxamide ribonucleotide (AICAR), a substance that activates AMPK, resulted in increased autophagosome accumulation when compared to using liraglutide alone. Flow cytometry analysis showed that liraglutide increased early apoptosis levels in a dose-dependent way, and when combined with AICAR, early apoptosis levels were further boosted. They also explored the links between immunohistochemical staining of GLP-1R and clinical characteristics, discovering that endometrial cancer tissues showed GLP-1R expression and that elevated GLP-1R levels were linked to improved progression-free survival (PFS). Furthermore, strong expression of GLP-1R was significantly associated with a favorable hormone receptor status and a lower histological grade [11]. 

In 2021, Zhu and colleagues found that the GLP-1 analog exenatide had a notable effect on decreasing endometrial damage and fibrosis in rats. These results indicate that exenatide could potentially help in avoiding endometrial complications in diabetic women. A thorough examination of eight trials showed that exenatide was not as efficient as liraglutide in controlling blood glucose levels and aiding in weight reduction. Based on these findings, the researchers set out to determine if liraglutide can also prevent the growth of EC cells and increase the levels of progesterone (PGR), ultimately reducing progesterone resistance in EC cells. Their research discovered that in Ishikawa and HEC-1B EC cells, liraglutide successfully inhibited the growth of EC cells in a manner that depended on both time and concentration. Moreover, liraglutide was discovered to enhance the PGR expression and effectively inhibit the growth of EC cells in cooperation with medroxyprogesterone acetate (MPA). This indicates that liraglutide might increase sensitivity to progesterone, suggesting that combining liraglutide with MPA could be a possible treatment for precancerous lesions and EC in the future [13].

In 2022, Li and colleagues published their study on how GLP-1R suppresses the progression of endometrial carcinoma by activating the cyclic adenosine monophosphate/protein kinase A (cAMP/PKA) pathway. Their research showed a noticeable drop in GLP1R levels in EC cells, suggesting that the tumor-suppressive role of GLP1R in EC may be mediated through the cAMP/PKA pathway. The cAMP/PKA pathway is a widely recognized cell signaling pathway that involves G-protein-coupled receptors. External signaling molecules stimulate membranous bodies, activating adenylyl cyclase in cells. This leads to an elevated cAMP level, which then activates PKA to initiate various biological reactions. This pathway has also been demonstrated to play a role in cell growth, differentiation, programmed cell death, ion movement, control of metabolic processes, and activation of genes. Analysis of bioinformatics data showed a direct correlation between the levels of GLP1R and PKA expression. The significant rise in cAMP levels in endometrial cancer cells was a result of the heightened expression of GLP1R. On the other hand, inhibition of PKA decreased cellular cAMP levels and reversed the increase in cAMP caused by overexpression of GLP1R. Likewise, transfection of GLP1R overexpression vector and si-PKA separately induced changes in GLP1R and p-PKA/PKA protein levels, while PKA silencing counteracted the impact of GLP1R overexpression. Moreover, tumor size and mass were reduced after the introduction of GLP1R overexpression vector, while they were increased following the introduction of si-PKA. Silencing of PKA reversed the growth inhibition of tumors caused by overexpressed GLP1R [14]. 

Li and colleagues have just released a new study that goes against the findings mentioned earlier. Their discoveries suggest that GLP1R not only boosts the viability, movement, and penetration of EC cells, but also offers defense against ferroptotic cell demise. The research noted a notable rise in GLP1R messenger RNA (mRNA) levels in EC tissues compared to normal tissues, along with an increase in GLP1R at the protein level in tumor tissues compared to normal tissues. Additionally, the data indicated that inhibiting GLP1R resulted in a significant reduction in cell survival over time. The research also investigated how the inhibition of GLP1R affects apoptosis in EC cells acanthosis nigricans 3rd attempt-carcinoma (AN3CA) and observed a decreased rate of apoptosis in AN3CA cells treated with a GLP-1 agonist compared to the control group. An evaluation of AN3CA cells showed a notable rise in migration distance when compared to the control group. Furthermore, the GLP-1 agonist notably boosted the quantity of invasive cells, suggesting that the up-regulation of GLP1R actively encourages the migration and invasion abilities of EC cells. To sum up, the research indicates that increasing GLP1R expression could potentially safeguard EC cells against ferroptotic cell demise [15].

GLP-1RAs and prostate cancer

In 2020, Shigeoka and colleagues conducted a study on the correlation between GLP-1 RAs and prostate cancer. In the research, they showed that the presence of GLP-1R in human prostate cancer cells was inversely associated with cancer growth and that increasing GLP-1R levels inhibited the growth of prostate cancer cells by slowing down cell division both in laboratory settings and in living organisms. The level of GLP-1R expression showed an inverse correlation with both the Gleason score and the growth of prostate cancer in human prostate cancer. This information suggests that the presence of GLP-1R could indicate early-stage prostate cancer, and using a GLP-1RA could be a treatment for patients with type 2 diabetes mellitus and early-stage prostate cancer complications [16].

GLP-1RAs and cervical cancer

Inflammation plays a typical role in the development of diabetes and cancer, particularly in virus infection-related cancers like human papillomavirus (HPV)-16 and -18 induced cervical cancer. Mao and colleagues discovered that prostate specific membrane antigen 2 (PSMA2), a gene found in the proteasome, plays a proto-oncogenic role in promoting the growth of cervical cancer cells. This suggests its potential proto-oncogenic role in cervical cancer samples from T2DM patients, supported by evidence from cell and animal studies. They discovered an elevated co-expression of GLP-1R and PSMA2 in cervical cancer models, both in humans and experiments, that could be reduced by exendin-4. The data from proteasome inhibitors and GLP-1 mimetics, currently in clinical use, have significant translational potential for patients with both T2DM and cancer, who commonly exhibit co-expression of PSMA2 and GLP-1R. Clinical trials should investigate the potential anti-cancer impact of exendin-4 in these individuals [17].

GLP-1RAs and lung cancer

Pu and his team researched how the glucagon-like-peptide-1 receptor agonist, liraglutide, affects lung cancer and its possible benefits in preventing lung aging induced by high glucose levels. The researchers observed a significant decrease in the proliferation, cell cycle, and migration of lung cancer cells after liraglutide treatment in comparison to the control group. Furthermore, liraglutide was demonstrated to block the epithelial-mesenchymal transition in lung cancer cells in comparison to the control group. Experiments conducted in living organisms also showed that liraglutide can inhibit the growth of lung cancer. The research findings suggested that liraglutide has the potential to decrease the aging process in the lungs. Thus, the researchers deduced that liraglutide has both anti-cancer effects and helps in reducing lung damage [18].

GLP-1RAs and pancreatic cancer

Glucagon-like peptide-1 stimulates the strengthening of pancreatic β-cells by activating the GLP-1 receptor. The possible connection between this action and the expansion of the exocrine pancreas, which may result in a higher chance of developing pancreatic cancer, has sparked controversy [19]. Different clinical data and experiments with animals have shown contradictory results. Current research shows that both liraglutide and exenatide therapies are linked to an increased likelihood of developing pancreatic cancer. Prolonged use of GLP-1RAs has also been associated with increased serum lipase and amylase levels in numerous type-2 diabetes patients, indicating potential pancreatic injury and inflammation. Inflammation or tissue injury can play a role in the development of cancer by changing the behavior of acinar and endocrine cells, resulting in the growth of ductal cells and the creation of early stage lesions that have the potential to develop into pancreatic cancer in the future. On the other hand, research conducted by Zhao et al. in 2014 found that liraglutide hinders cell growth and prompts cell death in human pancreatic cancer cells in a lab setting, while also decreasing the size of pancreatic tumors in animal models [20]. The impacts rely on GLP-1R and are facilitated by cAMP activation, leading to the inhibition of Akt and extracellular signal-regulated kinase (Erk) signaling. Moreover, in individuals suffering from pancreatic cancer, tumors that exhibit GLP-1R are notably smaller in size compared to those that do not express GLP-1R, indicating a possible reduction in tumor size due to GLP-1R signaling. In 2016, Marco Dal Molin and colleagues discovered that normal and neoplastic pancreatic cells have lower levels of GLP-1R in comparison to non-neoplastic pancreatic cells. [21]. Cao et al. came to the same conclusions in their meta-analysis [22].

GLP-1RAs and thyroid cancer

Multiple preclinical experiments on mice given exenatide or liraglutide showed an increased incidence of C-cell neoplasia and thyroid tumor formation. Extended use of exenatide or liraglutide was associated with a notable increase in plasma calcitonin levels, and C-cell hyperplasia is viewed as a pre-malignant state culminating in thyroid C-cell in situ carcinoma. However, the most recent meta-analysis for administering semaglutide has not documented any significant risk for thyroid cancer [23]. According to current state of knowledge, it is still not advised to prescribe GLP-1 agonists for individuals with a personal or family history of medullary thyroid cancer (MTC) or multiple endocrine neoplasia type-2 [24].

GLP-1RAs and chemotherapy

An additional potential role of studying GLP-1R is to understand how it affects how human endometrial cancer cells respond to chemotherapy when in high glucose conditions. Chemotherapy is an essential cancer treatment, and multidrug resistance (MDR) is a key element that can cause treatment to fail, tumors to advance, or cancer to relapse. The causes of drug resistance in cancer are intricate, with hyperglycemia believed to contribute to resistance to chemotherapy. The extent to which hypoglycemic agents can offset the adverse impacts of elevated glucose levels on the effectiveness of chemotherapy remains largely uncertain. Hence, Zhang and her team conducted research to assess how the anti-diabetes drug exendin-4, a GLP-1 receptor agonist, affects human endometrial cancer cells' reaction to chemotherapy following extended exposure to elevated glucose levels. This study examined Ishikawa and HEC1B, two frequently utilized cell lines of human endometrial adenocarcinoma. The findings showed that high blood sugar levels may provide protection to cancer cells against the damaging effects of cisplatin (DDP), but this protection was reversed by exendin-4. Additionally, exendin-4 reversed the pro-apoptotic effects of DDP in both cell lines that were counteracted by hyperglycemia. Furthermore, exendin-4 countered the effects of high glucose by boosting the cell count in the S phase following DDP treatment in high glucose environments. The research also indicated that exendin-4 was able to undo the impacts of elevated glucose on these cells by activating AMPK signaling, which controls the reactive oxygen species (ROS)-related mitochondrial pathway of programmed cell death. These results reveal the molecular pathways implicated in chemoresistance caused by high blood sugar levels and suggest that the anti-diabetic medication exendin-4, which activates the GLP-1R receptor, could potentially combat this resistance in endometrial cancer [25].

GLP-1RAs and other diseases

Zhao et al. gave a thorough analysis in their 2021 study about how GLP-1RAs affect different human organs such as the pancreas, heart, gut, liver, and brain. More precisely, GLP-1RAs have been demonstrated to stimulate the formation of new β-cells and increase insulin synthesis, as well as participate in the control of autophagy and enhancement of lipid profiles. Moreover, these agents also demonstrate cardioprotective effects by improving heart muscle contraction and uptake of glucose, while reducing damage from lack of blood flow. When it comes to liver health, GLP-1RAs may help decrease liver fat, plasma liver enzymes, and steatosis. Additionally, they have been associated with enhancements in synaptic plasticity, nerve differentiation, and the protection against various neurological disorders like Parkinson's and Alzheimer's diseases, stroke, and other conditions (Table 1) [20].

**Table 1:** Effects of GLP-1RAs on multiple human organs GLP-1RAs: Glucagon-like peptide-1 receptor agonists The upstream arrows indicate the increase while the downstream arrows indicate the decrease of the impact of GLP-1RAs in target organs [20].

Target organ	GLP-1RAs
Brain	Neuroprotection
	↑Neurogenesis
	↑Memory
Liver	↑Glucogen storage
Fat cells	↑Glucose uptake
	↑Lipolysis
Kidney	↑Natriuresis
Blood vessel	↑Endothelium-dependent vasodilation
Skeletal muscle	↑Glucose uptake
Pancreas	↑New β-cell formation
	↓β-cell apoptosis
	↑Insulin biosynthesis
Heart	↑Myocardial contractility
	↑Heart rate
	↑Myocardial glucose uptake
	↓Ischemia-induced myocardial damage

Future perspectives

Progesterone is a frequently utilized, safe, and effective hormone oral treatment for young patients with IA stage, absence of muscle infiltration, and PGR-positive EC [26]. Nevertheless, progesterone resistance is commonly seen in clinical settings. Hence, it is essential to find new agents that can increase PGR expression to enhance the effectiveness of hormone therapy with these drugs. Multiple research studies have concentrated on metabolic syndrome, specifically obesity and T2DM, as potential risk factors for EC. Providing treatment with efficient drugs for metabolic syndrome (MS) has been proven to lower the onset of EC and improve its outlook. The newly created medication liraglutide, which is an analog of GLP-1R and designed for treating diabetes, presents a new way to address MS. Despite its common usage for obesity and type 2 diabetes, its application for cancer patients is restricted and its exact molecular process is still unknown [27]. Therefore, it is advised to start a randomized trial of oral progestins/LNG-IUD with or without adding GLP-1 analogs [28]. Endometrial cancer is closely linked to obesity. Several studies have shown that losing 5% of body weight over a three-year period can lead to a 39% reduction in the risk of developing endometrial cancer. Interestingly, women with obesity experience the most benefits from weight loss, but women with a normal BMI also see positive effects. Bariatric surgery changes the structure of the digestive system by limiting stomach size, decreasing nutrient uptake, and causing quick feelings of fullness. Bariatric surgery is the most successful method for fighting obesity, leading to a permanent decrease in weight of around 29 kg, depending on the type of surgery performed. A study combining results has indicated that this method successfully decreases the likelihood of EC by 62%. Bariatric surgery, despite its potential to prevent EC, is not frequently carried out in clinical settings because of long-term risks such as malabsorption, nutritional deficiencies, and dumping syndrome [7].

## Conclusions

Endometrial cancer is becoming more common in young nulliparous women who desire to become pregnant, although the incidence remains low. Scientific data indicates that Grade 1 endometrioid endometrial cancer at an early stage can be safely and effectively treated with conservative methods. Nevertheless, this therapy may not be appropriate for every woman and should be considered on a case-by-case basis following a comprehensive medical assessment, imaging, hysteroscopic evaluation, and expert histological diagnosis. Patients who closely follow the treatment plan have a higher chance of attaining a successful pregnancy. The majority of individuals with endometrial hyperplasia or cancer are overweight. This, combined with polycystic ovarian syndrome and T2DM, can collectively raise the likelihood of developing endometrial cancer. The levonorgestrel intrauterine device is commonly utilized by patients wanting to preserve their fertility. If initial progestin treatment is ineffective, there is currently no successful alternative therapy available. Customized treatment strategies are necessary if initial progestin therapy is unsuccessful.

Glucagon-like peptide-1 receptor agonists, a type of diabetes medication, could potentially aid in preserving fertility for obese patients with endometrial cancer by lowering body mass index, inflammation, and insulin resistance, hence increasing likelihood of future pregnancy. These compounds have demonstrated hopeful results in stopping cancer cell growth, promoting programmed cell death, and controlling blood vessel formation, and have been found to be advantageous in different types of cancers. Nevertheless, there are concerns about the possibility of causing cancer, since liraglutide has been linked to a higher rate of thyroid and pancreatic cancers. Although they show promise in oncology, limited use in clinical settings, lack of knowledge, and the necessity for more research persist.
